# Supplementary material for: Genetics of cardiometabolic disease progression
Source: medRxiv. 2025 Feb 3:2025.02.01.25321518. Preprint. [Version 1] doi: 10.1101/2025.02.01.25321518 (PMC11838626; doi:10.1101/2025.02.01.25321518)
Supplement: Supplement 1 [file NIHPP2025.02.01.25321518v1-supplement-1.pdf]

medRxiv preprint doi: https://doi.org/10.1101/2025.02.01.25321518; this version posted February 3, 2025. The copyright holder for this preprint (which was not certified by peer review) is the author/funder, who has granted medRxiv a license to display the preprint in perpetuity.
It is made available under a CC-BY 4.0 International license .

Supplementary tables:

Supplementary Table 1. Overview of surgical phenotypes generated from multiple OPCS-4 codes from in-hospital records in UK Biobank.

Name	Characteristics	OPCS-4 codes
Percutaneous Coronary intervention (angioplasty)		K491 K492 K493 K494 K498 K499 K501 K502 K503 K504 K508 K509 K751 K752 K753 K754 K758 K759
Coronary artery bypass grafting (CABG)	A procedure to improve poor blood flow to the heart. CABG uses blood vessels from another part of the body and connects them to blood vessels above and below the narrowed artery, bypassing the narrowed or blocked coronary arteries.	K401 K402 K403 K404 K408 K409 K411 K412 K413 K414 K418 K422 K424 K431 K433 K439 K441 K442 K448 K449 K451 K452 K453 K454 K455 K456 K458 K459 K461 K462 K463 K464 K469
Carotid artery surgery		L291 L293 L294 L295 L297 L298 L299 L301 L302 L303 L304 L305 L308 L309 L311 L313 L314 L318 L319 O121 O122 O128 O129

Abdominal aortic aneurysm		L181 L182 L183 L184 L185 L186 L188 L189 L191 L192 L193 L194 L195 L196 L198 L199 L271 L272 L273 L274 L275 L276 L278 L279 L281 L282 L283 L284 L285 L286 L288 L289
Femoral/Iliac aneurysm		L491 L492 L493 L495 L496 L498 L499 L511 L512 L513 L514 L515 L516 L518 L519 L521 L522 L528 L531 L532 L533 L538 L539 L541 L542 L544 L548 L549 L571 L572 L573 L575 L591 L592 L593 L594 L595 L596 L597 L598 L599 L601 L602 L603 L604 L608 L609 L622 L623 L624 L628 L629 L631 L632 L635 L638 L639
Embolectomy	Removal of blood clot	L124 L131 L253 L263 L303 L343 L383 L392 L421 L432 L461 L532 L542 L622 L632 L701 L712

Peripheral artery interventions		L181 L182 L183 L184 L185 L186 L188 L189 L191 L192 L193 L194 L195 L196 L198 L199 L201 L204 L205 L206 L208 L211 L212 L213 L215 L216 L218 L231 L232 L233 L234 L235 L236 L238 L239 L251 L252 L253 L254 L255 L258 L259 L261 L262 L265 L266 L267 L268 L269 L271 L272 L273 L274 L275 L276 L278 L279 L281 L282 L283 L284 L285 L286 L288 L289 L291 L293 L294 L295 L297 L298 L299 L301 L302 L303 L304 L305 L308 L309 L311 L313 L314 L318 L319 L371 L373 L378 L381 L382 L383 L384 L388 L412 L413 L416 L418 L419 L423 L424 L428 L431 L432 L435 L438 L439 L451 L452 L463 L464 L468 L471 L474 L478 L491 L492 L493 L495 L496 L498 L499 L501 L503 L505 L506 L508 L509 L511 L512 L513 L514 L515 L516 L518 L519 L521 L522

		L528 L531 L532 L533 L538 L539 L541 L542 L544 L548 L549 L561 L562 L563 L571 L572 L573 L575 L578 L581 L582 L583 L584 L585 L587 L588 L589 L591 L592 L593 L594 L595 L596 L597 L598 L599 L601 L602 L603 L604 L608 L609 L622 L623 L624 L628 L629 L631 L632 L635 L638 L639 L661 L662 L663 L664 L665 L667 L701 L702 L704 L705 L708 L709 L711 L712 L714 L715 L716 L717 L718
Pacemaker	A pacemaker (artificial pacemaker surgery) is a small device that is placed under the chest skin below the collarbone. One or two wires connect the pacemaker to the heart chambers. It generates a small electrical current that	K601 K605 K606 K607 K611 K615 K616 K617 K618 K619

	stimulates the heart muscle and ensures regular pumping activity.	
Diabetes related eye surgery		C821 Y084
Amputation	Amputation of leg, foot or toe	X091 X092 X093 X094 X095 X098 X101 X102 X103 X104 X108 X109 X111 X112 X118 X119
End stage kidney disease surgery		X401 X402 X403 X404 X405 X406 X408 X409 M011 M012 M013 M014 M015 M018 M019
Liver surgery		J011 J012 J013 J015 J018 J019 J021 J022 J023 J024 J025 J026 J027 J028 J029 J031 J032 J033 J034 J035 J038 J039
Cardioversion	Restore abnormal heart rhythm (arrhythmia) by sending electrical signals to the heart through electrodes	X501 X502

	placed on the chest.	
Percutaneous radiofrequency catheter ablation for atrial fibrillation		K574 K621 K622 K623

All OPCS-4 are listed on the UK Biobank website: https://biobank.ctsu.ox.ac.uk/crystal/field.cgi?id=41272

https://www.nice.org.uk/Media/Default/About/what-we-do/NICE-guidance/NICE-interventional-procedures/OPCS-reports-September-2012.pdf

medRxiv preprint doi: https://doi.org/10.1101/2025.02.01.25321518; this version posted February 3, 2025. The copyright holder for this preprint (which was not certified by peer review) is the author/funder, who has granted medRxiv a license to display the preprint in perpetuity.
It is made available under a CC-BY 4.0 International license .

Supplementary Table 2. Number of white British and non-British white individuals having the initial disease outcome in UK Biobank.

Disease phenotype outcomes - ICD-10	White British cases	Non-British white cases
Essential (primary) hypertension - I10	83,727	
Disorders of lipoprotein metabolism and other lipidemias (hyperlipidemia)- E78	68,477	
Chronic ischaemic heart disease - I25	25,378	
Obesity	22667	
Angina pectoris - I20	20,542	
Non-insulin-dependent diabetes mellitus (T2D) - E11	20,475	
Atrial fibrillation and flutter - I48	17,166	

medRxiv preprint doi: https://doi.org/10.1101/2025.02.01.25321518; this version posted February 3, 2025. The copyright holder for this preprint (which was not certified by peer review) is the author/funder, who has granted medRxiv a license to display the preprint in perpetuity.
It is made available under a CC-BY 4.0 International license .

Supplementary table 3. Phenotypes included as disease outcomes (secondary diagnosis) for white British individuals in UK Biobank

Phenotype	ICD-10	White British cases
Disease phenotype outcomes - ICD-10		
Essential (primary) hypertension	I10	83727
Disorders of lipoprotein metabolism and other lipidemias	E78	68477
Chronic ischaemic heart disease	I25	25378
Obesity	E66	22667
Angina pectoris	I20	20542
Non-insulin-dependent diabetes mellitus (T2D)	E11	20475
Atrial fibrillation and flutter	I48	17166
Acute myocardial infarction	I21	12514
Chronic renal failure	N18	11928
Peripheral artery disease	I73	7014
Heart failure	I50	6812
Other cardiac arrhythmias	I49	6339
Acute renal failure	N17	6259
Stroke, not specified as haemorrhage or infarction	I64	5918

medRxiv preprint doi: https://doi.org/10.1101/2025.02.01.25321518; this version posted February 3, 2025. The copyright holder for this preprint (which was not certified by peer review) is the author/funder, who has granted medRxiv a license to display the preprint in perpetuity.
It is made available under a CC-BY 4.0 International license .

Pulmonary embolism	I26	5890
Other diseases of liver	K76	5839
Cerebral infarction	I63	3907
Acute pancreatitis	K85	2440
Unspecified renal failure	N19	2034
Aortic aneurysm and dissection	I71	1983
Other disorders of arteries and arterioles	I77	1631
Cardiomyopathy	I42	1572
Atherosclerosis	I70	1481
Ulcer of lower limb	L97	1453
Other inflammatory liver diseases	K75	1309
Hypertensive renal disease	I12	1289
Fibrosis and cirrhosis of liver	K74	1192
Cardiac arrest	I46	1178
Intracerebral hemorrhage	I61	942
Hepatic failure, not elsewhere classified	K72	472
Hypertensive heart disease	I11	408

medRxiv preprint doi: https://doi.org/10.1101/2025.02.01.25321518; this version posted February 3, 2025. The copyright holder for this preprint
(which was not certified by peer review) is the author/funder, who has granted medRxiv a license to display the preprint in perpetuity.
It is made available under a CC-BY 4.0 International license .

Supplementary table 4. Phenotypes included as operation outcomes (surgeries performed after initial diagnosis) for white British individuals in UK Biobank

Operational phenotypes - OPCS-4	N British white	Non British White
Percutaneous Coronary intervention (angioplasty)	12620	
Coronary artery bypass grafting (CABG)	5770	
Carotid artery surgery	1428	
Cardioversion	4868	
Percutaneous radiofrequency catheter ablation for atrial fibrillation	1631	
Peripheral artery interventions		
Femoral/Iliac aneurysm		

medRxiv preprint doi: https://doi.org/10.1101/2025.02.01.25321518; this version posted February 3, 2025. The copyright holder for this preprint (which was not certified by peer review) is the author/funder, who has granted medRxiv a license to display the preprint in perpetuity.
It is made available under a CC-BY 4.0 International license .

Pacemaker	5710	
Diabetes related eye surgery	1533	
Amputation	970	
End stage kidney disease surgery	1497	
Liver surgery	782	

medRxiv preprint doi: https://doi.org/10.1101/2025.02.01.25321518; this version posted February 3, 2025. The copyright holder for this preprint (which was not certified by peer review) is the author/funder, who has granted medRxiv a license to display the preprint in perpetuity.
It is made available under a CC-BY 4.0 International license .

Supplementary Table 5. Time-to-event summary hazards ratio per standard deviation of the polygenic hazards score for all individuals and top 1, 5 and 10% and bottom 10% compared to 40-60%.

Polygenic Hazards Score by percentile for hyperlipidemia to coronary artery bypass graft	Hazards Ratio	*p*-value
All	1.3	4.5×10^{-9}
Top 1%	2.6	0.002
Top 5%	1.6	0.02
Top 10%	1.7	0.004
Bottom 10%	0.57	0.01

medRxiv preprint doi: https://doi.org/10.1101/2025.02.01.25321518; this version posted February 3, 2025. The copyright holder for this preprint (which was not certified by peer review) is the author/funder, who has granted medRxiv a license to display the preprint in perpetuity.
It is made available under a CC-BY 4.0 International license .

Supplementary table 6. Phewas (p <= 1x10^{-3}) for *LMO1* rs11041816 white British individuals from UK Biobank

Phenotype name	Cases	Beta	SE	*p*
Basal_metabolic_rate	331443	-0.0097896	0.00159998	9.45E-10
Trunk_predicted_mass	331144	-0.0093316	0.00152599	9.66E-10
Whole_body_fat-free_mass	331427	-0.0092592	0.00152996	1.43E-09
Trunk_fat-free_mass	331175	-0.009255	0.00153116	1.50E-09
Leg_predicted_mass_(left)	331390	-0.0093417	0.00156475	2.37E-09
Leg_fat-free_mass_(left)	331395	-0.0093907	0.00157524	2.50E-09
Whole_body_water_mass	331451	-0.0091062	0.00153289	2.84E-09
Leg_fat-free_mass_(right)	331421	-0.0090076	0.0015668	8.98E-09
Leg_predicted_mass_(right)	331421	-0.0087663	0.00155643	1.78E-08
Arm_fat-free_mass_(left)	331299	-0.0085998	0.0015466	2.69E-08
Arm_predicted_mass_(left)	331286	-0.0085342	0.00153917	2.95E-08
Arm_predicted_mass_(right)	331354	-0.0081297	0.00150829	7.05E-08
Weight	331454	-0.011343	0.00216126	1.54E-07
Arm_fat-free_mass_(right)	331359	-0.0078944	0.00151458	1.87E-07
Weight	336211	-0.0110263	0.00214749	2.83E-07
Sitting_height	336453	-0.0086178	0.00183921	2.79E-06
Standing_height	336442	-0.0077758	0.00171254	5.61E-06
Albumin	291507	-0.0114847	0.00263346	1.29E-05

medRxiv preprint doi: https://doi.org/10.1101/2025.02.01.25321518; this version posted February 3, 2025. The copyright holder for this preprint (which was not certified by peer review) is the author/funder, who has granted medRxiv a license to display the preprint in perpetuity.
It is made available under a CC-BY 4.0 International license .

Number_of_treatments/medications_taken	337100	-0.0092411	0.00216915	2.04E-05
Forced_expiratory_volume_in_1-second_(FEV1),_predicted	110404	-0.0077084	0.00182057	2.30E-05
Impedance_of_leg_(left)	331712	0.0092624	0.00223515	3.41E-05
Impedance_of_whole_body	331700	0.0074495	0.0018676	6.64E-05
Vascular/heart_problems_diagnosed_by_doctor_High_blood_pressure	92713	-0.0221454	0.00569528	0.00010093
Arm_fat_mass_(left)	331303	-0.0091815	0.00239404	0.00012552
Arm_fat_mass_(right)	331363	-0.009166	0.00239174	0.00012694
Calcium	291834	-0.0100802	0.00263176	0.00012806
Whole_body_fat_mass	330911	-0.0090973	0.00238385	0.00013554
Number_of_self-reported_non-cancer_illnesses	337100	-0.0082457	0.00219593	0.00017339
Urate	318517	-0.0094056	0.0025198	0.00018948
TTE_other_noninflammatory_disorders_of_uterus,_except_cervix	5035	0.04787546	0.0129217	0.00021108
TTE_congenital_malformations_of_posterior_segment_of_eye	171	0.40022473	0.109064	0.00024293
Leg_fat_mass_(right)	331429	-0.0071641	0.00195337	0.00024492
TTE_essential_(primary_hypertension)	83779	-0.0216088	0.00592368	0.00026445
Body_mass_index_(BMI)	331449	-0.0085767	0.00244233	0.00044534
Which_eye(s)_affected_by_amblyopia_(lazy_eye)_(Left_eye)	2431	0.16755473	0.04775	0.00044975
Trunk_fat_mass	331236	-0.0085777	0.00245448	0.00047467
respiratory_/_intrathoracic_cancer	3032	-0.0912987	0.0261255	0.00047471
TTE_sexual_dysfunction,_not_caused_by_organic_disorder_or_disease	1405	-0.1328585	0.0383319	0.00052827
Impedance_of_leg_(right)	331715	0.00761036	0.00221704	0.00059775

medRxiv preprint doi: https://doi.org/10.1101/2025.02.01.25321518; this version posted February 3, 2025. The copyright holder for this preprint (which was not certified by peer review) is the author/funder, who has granted medRxiv a license to display the preprint in perpetuity.
It is made available under a CC-BY 4.0 International license .

Body_mass_index_(BMI)	336099	-0.0082379	0.00242609	0.00068497
Leg_fat_mass_(left)	331411	-0.0065259	0.00193143	0.00072811

Supplementary Table 7. Replication of significant PHS associations in non-British White individuals from UK Biobank (n=28,134)

Phenotypes	N cases	Scale HR	Scale P-value	Scale C
Hyperlipidemia to CABG	145	1.15	0.09	0.54
Hyperlipidemia to chronic ischaemic heart disease	595	1.16	$2.8x10^{-4}$	0.55
Hypertension to angioplasty	201	1.10	0.19	0.53
Hypertension to T2D	381	1.21	$2.40x10^{-4}$	0.55
Hyperlipidemia to myocardial infarction	276	1.10	0.09	0.54
Hyperlipidemia to angina	362	1.17	$2.9x10^{-3}$	0.54
Hyperlipidemia to angioplasty	237	1.06	0.39	0.52
Chronic ischaemic heart disease to CABG	217	1.14	0.05	0.54

Hypertension to CABG	121	1.19	0.06	0.55
Angina to CABG	158	1.12	0.17	0.53

Supplementary Figure 1. Phewas plot of rs4480535 upstream of *LMO1* shows associates with lower risk of CABG in Finngen.

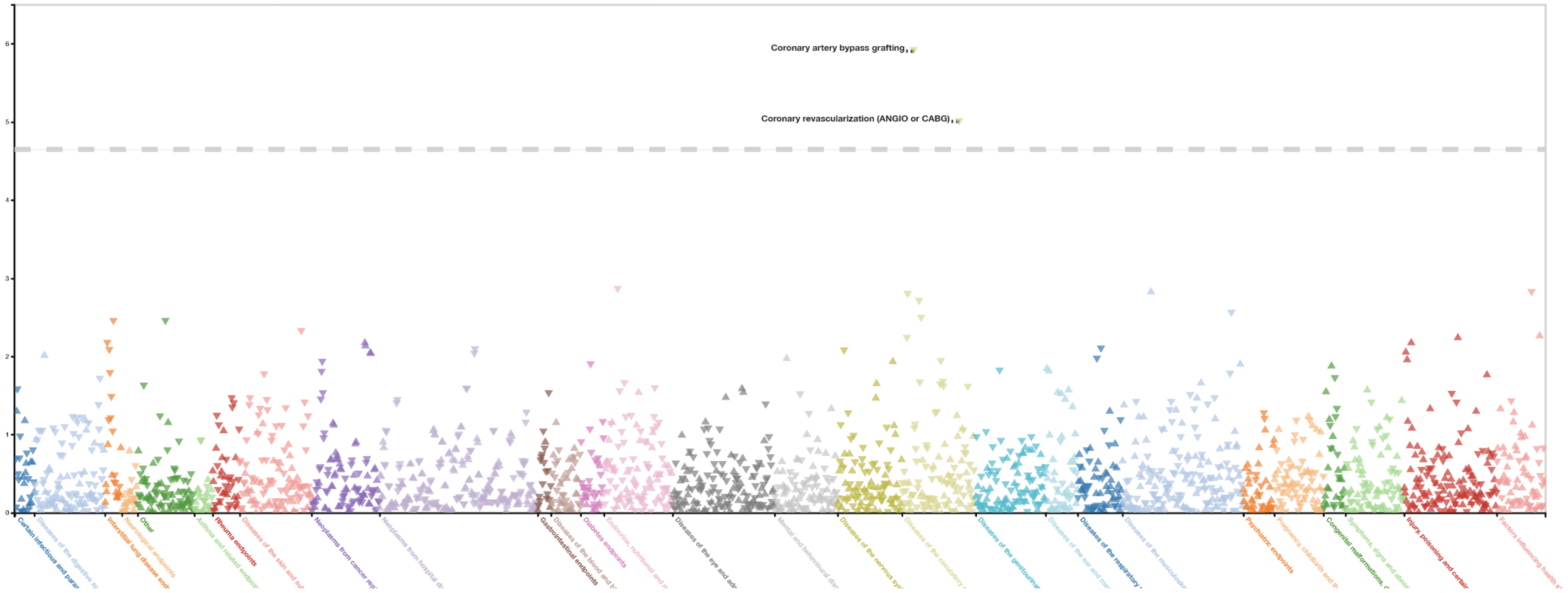

Finngen freeze 4. Variant rs4480535 (11:8302272:A:G) upstream of *LMO1* has a protective effect on coronary artery bypass grafting beta = -0.12, $p = 1.22 \times 10^{-6}$. Cases = 4449 and controls = 166928

http://r4.finngen.fi/region/I9_CABG/gene/LMO1

Supplementary figure 2. GTEx eQTL for variant rs11041816 identified two genes *RIC3* and *LMO1*

eQTLs of chr11_8222251_A_G_b38

Copy CSV

Search: Show 10 entries

Gencode Id	Gene Symbol	Variant Id	SNP		P-Value	NES	Tissue	Actions
ENSG00000166405.14	RIC3	chr11_8222251_A_G_b38	rs11041816	dbSNP	2.2e-7	0.13	Skin - Sun Exposed (Lower leg)	eQTL violin plot, IGV Browser, Multi-tissue eQTL Plot
ENSG00000166407.13	LMO1	chr11_8222251_A_G_b38	rs11041816	dbSNP	0.000019	-0.084	Muscle - Skeletal	eQTL violin plot, IGV Browser, Multi-tissue eQTL Plot

Showing 1 to 2 of 2 entries

First Previous 1 Next Last
